# Exploring the Relationship between Skeletal Mass and Total Body Mass in Birds

**DOI:** 10.1371/journal.pone.0141794

**Published:** 2015-10-28

**Authors:** Elizabeth Martin-Silverstone, Orsolya Vincze, Ria McCann, Carl H. W. Jonsson, Colin Palmer, Gary Kaiser, Gareth Dyke

**Affiliations:** 1 Ocean and Earth Sciences, University of Southampton, Southampton, Hampshire, United Kingdom; 2 School of Earth Sciences, University of Bristol, Bristol, United Kingdom; 3 MTA-DE “Lendület” Behavioural Ecology Research Group, Department of Evolutionary Zoology, University of Debrecen, Debrecen, Hungary; 4 Faculty of Biology and Geology, Babes Bolyai University, Cluj Napoca, Romania; 5 Royal British Columbia Museum, Victoria, British Columbia, Canada; University of South Carolina, UNITED STATES

## Abstract

Total body mass (TBM) is known to be related to a number of different osteological features in vertebrates, including limb element measurements and total skeletal mass. The relationship between skeletal mass and TBM in birds has been suggested as a way of estimating the latter in cases where only the skeleton is known (e.g., fossils). This relationship has thus also been applied to other extinct vertebrates, including the non-avian pterosaurs, while other studies have used additional skeletal correlates found in modern birds to estimate TBM. However, most previous studies have used TBM compiled from the literature rather than from direct measurements, producing values from population averages rather than from individuals. Here, we report a new dataset of 487 extant birds encompassing 79 species that have skeletal mass and TBM recorded at the time of collection or preparation. We combine both historical and new data for analyses with phylogenetic control and find a similar and well-correlated relationship between skeletal mass and TBM. Thus, we confirm that TBM and skeletal mass are accurate proxies for estimating one another. We also look at other factors that may have an effect on avian body mass, including sex, ontogenetic stage, and flight mode. While data are well-correlated in all cases, phylogeny is a major control on TBM in birds strongly suggesting that this relationship is not appropriate for estimating the total mass of taxa outside of crown birds, Neornithes (e.g., non-avian dinosaurs, pterosaurs). Data also reveal large variability in both bird skeletal and TBM within single species; caution should thus be applied when using published mass to test direct correlations with skeletal mass and bone lengths.

## Introduction

Body mass (‘total body mass’; TBM) is strongly correlated with an animal’s biology, physiology and mode-of-life [1]. Thus, accurately measuring TBM allows other aspects of lifestyle to be inferred (e.g. metabolism, growth rate, population density, diet, reproductive strategy; [2–4]). TBM is the single most important variable controlling locomotor mode and hence biomechanics [5,6]. In birds, measuring and understanding body mass is critical because of flight: lift is directly proportional to TBM.

In living birds, TBM can be easily and accurately measured as the sum of skeletal and non-skeletal mass [7,8] by weighing living individuals or fresh carcasses. This relationship is relevant to the study of fossils because soft-tissues are never preserved in a useful form and because paleontologists have devoted a great deal of effort estimating TBM for extinct organisms (e.g. [8–12]). Establishing a robust and reliable relationship between TBM and its constituent skeletal mass provides a powerful tool for interpreting the fossil record. A previously published scaling exponent for extant birds [7] has, for example, been applied for mass estimation in another group of flying vertebrates, the extinct pterosaurs [13]; this is of interest because these vertebrates attained body sizes of approximately 250 kg and wingspans of 10–12 m, the largest flying animals ever [14].

Uncertainty in estimations of soft tissue densities in extinct animals [15] has meant that existing methods to calculate TBM in fossils either rely on body volume and/or density reconstructions (e.g.[10,12,16]) have been supplanted by the use of scaling relationships (e.g.[1,8,9,17]).

Prange et al. [7] presented the first comprehensive data-based study addressing the TBM/skeletal mass relationship in living birds and mammals. They reported correlations using measurements from literature (mammals) and from fresh bird carcasses and demonstrated an allometric relationship independent of ecology or lifestyle but similar for both groups. Substantial subsequent work has built on Prange et al. [7] employing allometric analyses to estimate the masses of extinct animals (e.g. [9,13,18]) and discussing the scaling relationships controlling avian body mass (e.g. [8,18–20]). However, most previous studies have one factor in common: although bone measurements come from skeletons, TBM is often derived from literature, most frequently the extensive compendium of Dunning [21] and it’s earlier iteration [22]. This means that while skeletal measurements capture individual and species-level variation, TBM almost never does. In addition, most of Dunning’s [21] species means for avian TBM, were themselves recycled from existing literature (e.g. [22]) and thus captured various subspecies and/or geographic regions within data offered for a single species. These factors are known to have a substantial affect TBM as avian body mass can vary significantly across a breeding range and with time of the year.

In this study, we revisit Prange et al. [7] presenting: (1) a re-analysis of this original dataset for birds; (2) analysis of a new unique dataset of individuals from a single museum collection; (3) exploration of a comprehensive pooled dataset comprising TBM and skeletal measurements from Prange et al. [7] augmented by a the new dataset; and (4) a study of whether additional factors such as ontogenetic stage, sex, or flight mode affect the TBM to skeletal mass relationship in birds. Importantly, TBM is a character of very high phylogenetic dependence [23], meaning that closely related species have similar body sizes. Therefore our analyses are controlled for common descent using a recent molecular phylogenetic tree for birds (see below). Finally, we comment on the implications of using scaling relationships for living birds to estimate TBM in extinct animals, especially with respect to the flight capabilities of birds and pterosaurs.

## Methods

### Data

The Prange et al. [7] dataset for living birds comprises 308 specimens encompassing 206 species. Our second dataset comprises skeletal element measurements and individual TBMs taken from birds in the ornithology collection at the Royal British Columbia Museum, Victoria, Canada (RBCM) (by GK and CJ), which is a public and permanent repository. Specimen numbers for all of the material studied can be found in the Supporting Information. This collection is unique because individual TBMs were recorded either when specimens were collected or when carcasses were donated to the RBCM. Thus, individual bones, skeletons, and TBMs for 487 extant flying birds in this collection were weighed and measured, encompassing 15 orders spanning 79 flying species (Supporting Information). In this data set, TBMs vary from 46g to 12000g. All skeletons were prepared at the RBCM for taxonomic evaluation; dry skeletal masses were measured using an electronic pan balance accurate to 0.1g. In cases where skeletons are not entirely complete because one of a pair of bones was broken or missing, the mass of the extant member of the pair was used. For completeness and for future studies, lengths and widths of individual bones were measured with electronic calipers accurate to 0.01mm (Supporting Information).

The RBCM dataset was also divided in order to test additional sources of variation. Sexual variation was explored by dividing the specimens into males and females, where known. Specimens where the sex was unknown were disregarded. Limited information was known on ontogenetic stage, allowing specimens to be divided only into hatchling year (HY), or above hatchling year (AHY). Differences between these two groups were then studied. Finally, flight mode was determined for each species. Each species was categorized into one of four flight modes, based on Close and Rayfield [24]: continuous flapping, flap-gliding, soaring, or burst-adapted flight. Flap-gliding refers to birds that interject periods of gliding (not soaring) between periods of flapping, at regular intervals (e.g. [24,25], while soaring specifically describes birds that use rising air or the wind shear over the sea (dynamic soaring) [26,27]. Burst-adapted flyers are those that typically stay on the ground (such as grouse or ptarmigans) but are capable of rapid take-offs with high frequency wing beats [24]. While prior studies have included intermittent bounding [24–26], which is typical of passerines, the RBCM dataset did not include any birds with this flight mode and it was therefore eliminated. Classification of flight mode was determined through the literature [24,27], by viewing video footage on Getty Images (www.gettyimages.co.uk), ARKive (www.arkive.org), The Internet Bird Collection (www.ibc.lynxeds.com), the Cornell Lab of Ornithology (www.macaulaylibrary.org), or in rare cases on YouTube (www.youtube.com). However, videos of some species were not found and flight mode for these species was assumed based on closely related species, or from personal observation.

Two significant outliers, almost certainly errors, in Prange et al. [7] were removed from our analyses: the Black-striped Sparrow (*Arremonops conirostris*) because its skeletal weight is listed as 50.5% of its TBM (the rest of the species in our data sets have a TBM range from 2.5% to 21.6%; Supporting Information) and the Pearl-spotted Owl (*Glaucidium perlatum*) because it's listed body weight is 6.9g (this species weighs on average 69g and 91g for males and females respectively; [21]). Additionally, the extinct Lord Howe Swamphen (*Porphyrio alba*) and *Rallus longirostris*, subject to taxonomic controversy, were removed, because their molecular taxonomic positions are unclear (e.g. [28,29]). Finally, Prange et al. [7] reported two subspecies of Sandhill Crane (*Grus canadensis*), of which we only used the one with a larger sample size (i.e. *tabida*) because phylogenetic comparative analyses used here only allow a single data point per species, while the two Sandhill Crane subspecies are considerably different [21], therefore calculating a single mean for the species may not be ideal. By removing these problematic taxa and individuals, the final Prange et al. [7] dataset re-analysed in this study consists of 300 specimens and 203 species.

### Analyses

We performed two initial sets of analyses. First, we re-analysed the bird data published by Prange et al. [7] using phylogenetic control (203 species) [30,31]. Then, we analysed the RBCM dataset both with and without phylogenetic control, as with the Prange et al. [7] dataset. After these initial analyses, we pooled the Prange et al. [7] data and the RBCM data and analysed this (270 species; 12 species overlap), using the same phylogenetic control. Average TBM and skeletal mass (SM) for overlapping species (n = 12) were calculated as weighted means, where weights were represented by the number of specimens measured in each collection. Finally, this method was then repeated for the RBCM dataset once it was divided into different sexes, ontogenetic stages, and flight modes. These different groups were then compared.

Since species are not independent data points (because of their shared evolutionary history [32]), we used phylogenetically controlled models for analyses. These models implement a variance-covariance matrix that helps to account for the expected similarity of species based on their degree of phylogenetic relatedness. For this, we used phylogenetic trees from http://birdtree.org [31]. We downloaded 1000 trees with the backbone tree of Hackett et al. [30] and calculated a dated, rooted, ultrametric consensus tree using the SumTrees software [33], and branch lengths calculated as arithmetic means of the lengths of the corresponding split or clade in the source trees. Both TBM and skeletal mass were log transformed (base 10) prior to analyses and association between skeletal mass and TBM was tested using phylogenetic generalised least squares (PGLS), as implemented in the R package caper [34]. We used skeletal mass as the response variable and TBM as the predictor variable (x) following Prange et al. [7] in order to compare the two results. Additionally, since these models are asymmetrical (i.e. the relationship between x and y depends on which one of these is the dependent and which is the explanatory variable) and the association had been repeatedly used to predict TBM from skeletal mass (incorrectly using the allometric equation of Prange et al. [7]), models were repeated with the two variable interchanged. Slope differences were tested using PGLS models where the dependent variable was expressed as SM minus SM predicted based on TBM, using the allometric equation published by Prange et al. [7]. In light of a recent critique of the major axis (e.g. RMA) regression approach used in estimating allometric associations [35,36], this approach was not used.

## Results and Discussion

**Fig 1:**
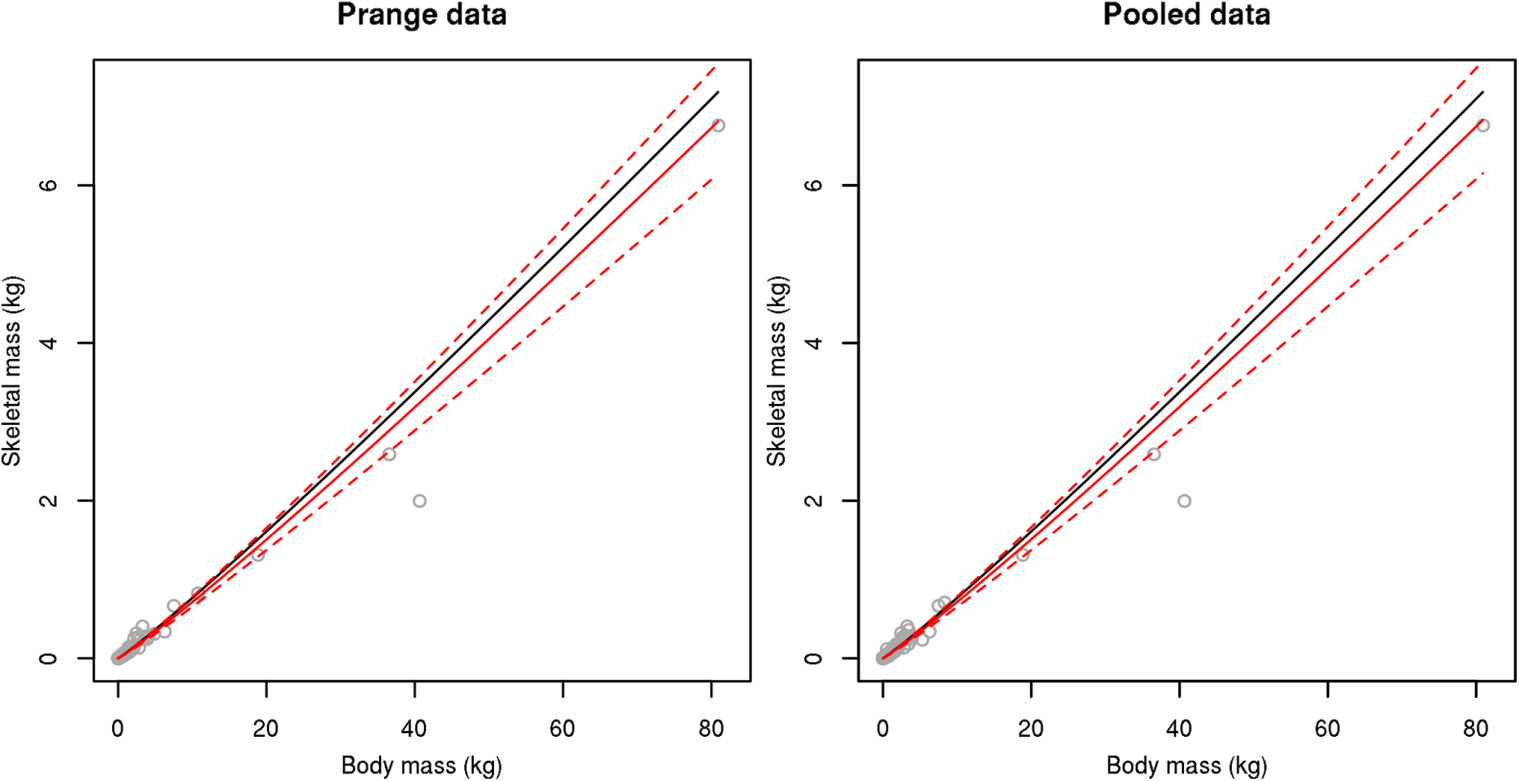
Linear scale association between total body mass and skeletal mass in birds. Plotted on a linear scale using (a) the data published by Prange et al. [7] and (b) the pooled dataset (RBCM + Prange et al. [7]). Black line represents the association estimated by Prange et al. [7], red line represents the association estimated here, dashed lines represent standard errors.

**Fig 2:**
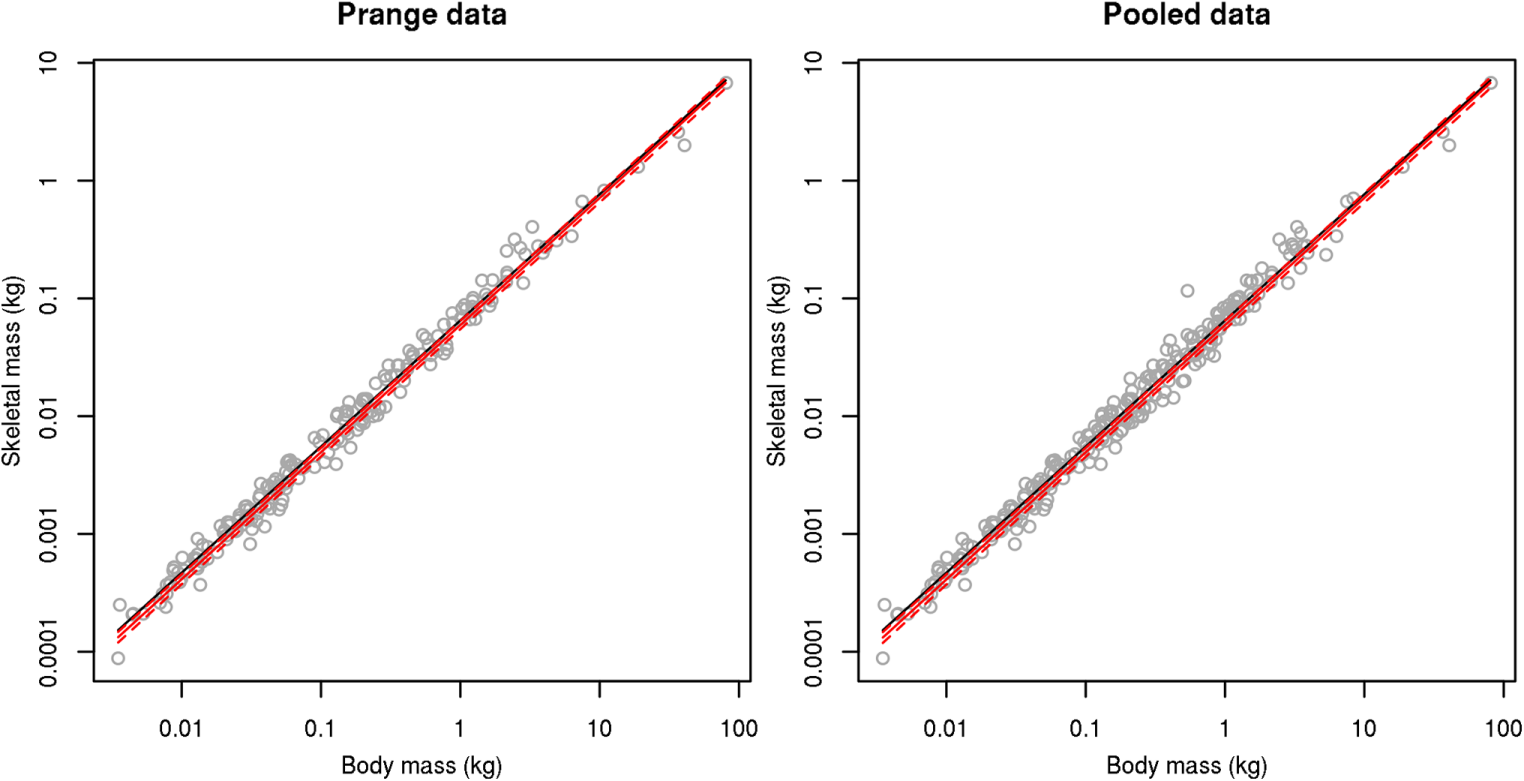
Logarithmic scale association between total body mass and skeletal mass in birds. Plotted on a log scale using (a) the data published by Prange et al. [7] and (b) the pooled dataset (RBCM + Prange et al. [7]). Black line represents the association estimated by Prange et al. [7], red line represents the association estimated here, dashed lines represent standard errors.

Our re-analysis of the Prange et al. [7] bird data using phylogenetic control found a slightly but not significantly larger allometric exponent than previously reported (skeletal mass, SM = 0.059 x TBM^1.079^, R^2^ = 0.97). The allometric exponent we estimate (1.079 ± 0.013 SE) does not differ significantly (t = 0.65, p = 0.5150) from the one estimated by Prange et al. ([7]: 1.071 ± 0.102) (Figs 1 and 2).

Analysis of our pooled dataset (n = 270 species, 787 specimens) also resulted in a slightly larger allometric exponent than estimated by Prange et al [7] (SM = 0.059 x TBM^1.082^, R^2^ = 0.97) (Figs 1 and 2). Again, our revised estimated allometric exponent (1.082 ± 0.012 SEM) does not differ significantly from Prange et al.’s [7] first estimate (t = 0.91, p = 0.3589). Additionally the allometric exponent (1.085 ± 0.029) in the equation calculated based on the RBCM dataset alone (SM = 0.064 x TBM^1.085^, R^2^ = 0.95) was similar to that estimated by Prange et al. [7] (t = 0.47, p = 0.6393). The reversed models, where we predicted TBM based on SM, resulted in an allometric equation of TBM = 12.560 x SM^0.901^ (SE = 0.011) for the data extracted from Prange et al. [7], and TBM = 12.395 x SM^0.895^ (SE = 0.010) for the pooled dataset and TBM = 10.601 x SM^0.871^ (SE = 0.023) for the RBCM dataset.

**Fig 3:**
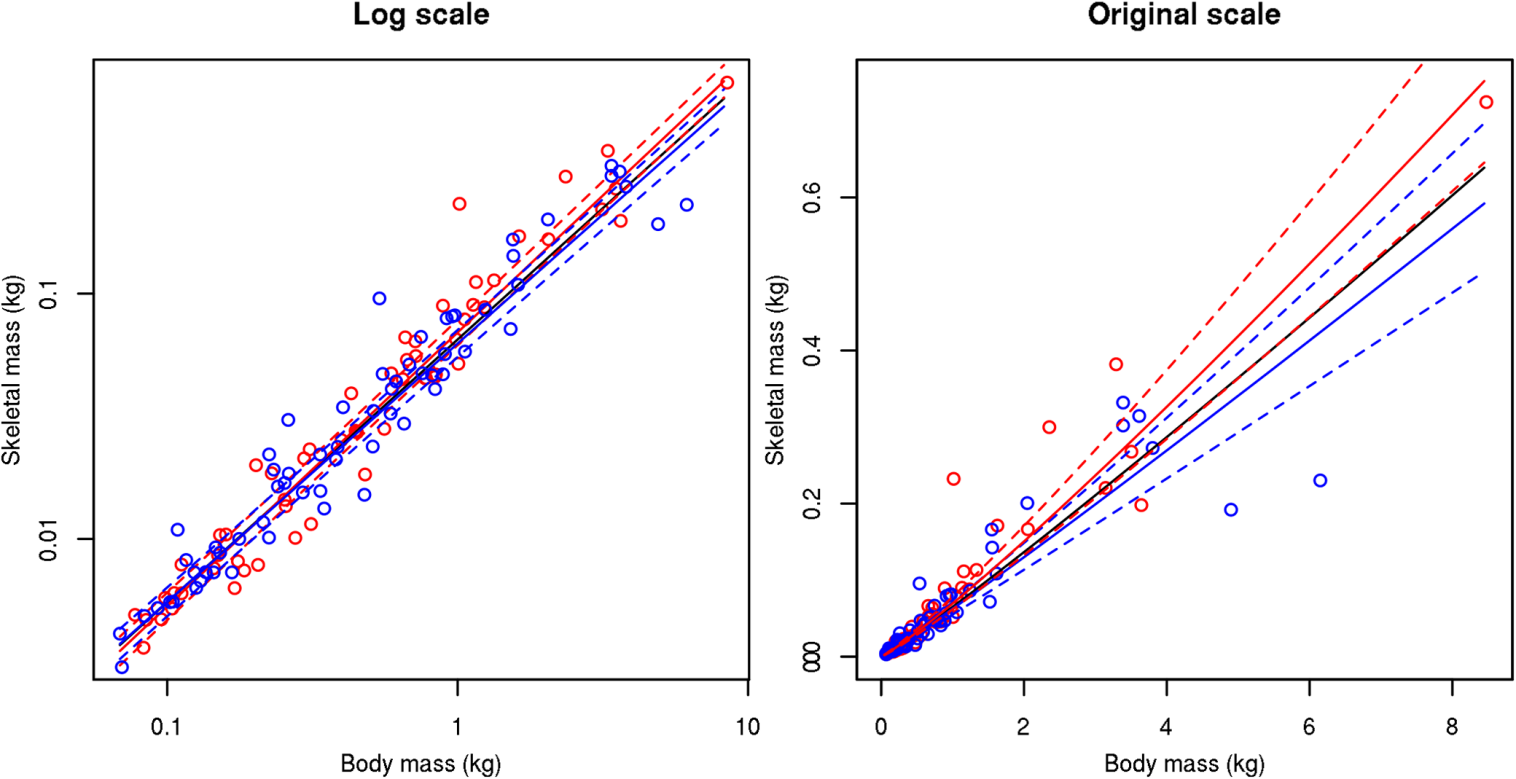
Sexual variation between total body mass vs. skeletal mass relationships in birds. Red = female, blue = male, black solid line represents the total relationship for the entire dataset, while coloured solid lines represent the association for each sex, dashed lines represent standard errors.

Analysis of sexual variation in the RBCM dataset of TBM vs. SM between females (58 species, SM = 0.070 x TBM^1.118^, SE = 0.035, R^2^ = 0.95) and males (64 species, SM = 0.063 x TBM^1.054^, SE = 0.036, R^2^ = 0.93) revealed no statistically significant difference in the allometric exponent between the two (t = 1.83, p = 0.07305) (Fig 3). Additionally, no statistically significant differences were found in the allometric exponent estimated based on specimens that were above their hatchling year (AHY, 77 species, SM = 0.064 x TBM^1.080^, SE = 0.029, R^2^ = 0.95) or in their hatchling year (HY, 17 species, SM = 0.090 x TBM^1.072^, SE = 0.086, R^2^ = 0.91), the only ontogenetic information available in the RBCM dataset (t = 0.29, p = 0.7746) (S1 Fig).

Finally, no statistical differences were found in the allometric exponent estimated based species with different flight modes of soaring (12 species, SM = 0.064 x TBM^1.045^, SE = 0.062, R^2^ = 0.97), flap-gliding (21 species, SM = 0.083 x TBM^1.153^, SE = 0.063, R^2^ = 0.95) and continuous flapping (43 species, SM = 0.067 x TBM^1.099^, SE = 0.040, R^2^ = 0.95) in the RBCM dataset. Difference in allometric exponents (flap-gliding–soaring, t = 1.63, p = 0.1206; flap-gliding–continuous, t = 0.76, p = 0.4579; continuous–soaring t = 1.19, p = 0.2395) (S2 Fig). Unfortunately, a model could not be made for the burst-adapted flyers as there were only three species, and therefore we could not test burst-adapted flyers against other flight modes.

Despite addition of considerably more, and arguably more accurate, mass data and analysis under phylogenetic control, our results corroborate the relationships reported in Prange et al. [7] (Figs 1 and 2). Thus, although we advocate use of our re-calculated scaling exponents, TBM and skeletal mass provide accurate proxies for estimating one another.

Nevertheless, comparing the new RBCM dataset with the original Prange et al. [7] dataset, several important further observations can be made. There is significant variation in the RBCM dataset, with many specimens deviating from the relationship (although overall well-correlated). This can be more easily seen when data is shown with a linear scale, rather than logarithmic (Fig 1) and can be explained mainly by two factors. Primarily, our new dataset is made up of a significant number of individuals from each species: the average number of specimens per species in the RBCM dataset is 12.97 (ranging from 1 to 30), while the Prange et al. [7] data averages 2.14 individuals per species (ranging from 1 to 6). With so many more individuals used here, further variation is expected as a larger subset of each species has been measured. The overall relationship, while very similar, has changed slightly; the RBCM dataset is also slightly less positively allometric.

**Fig 4:**
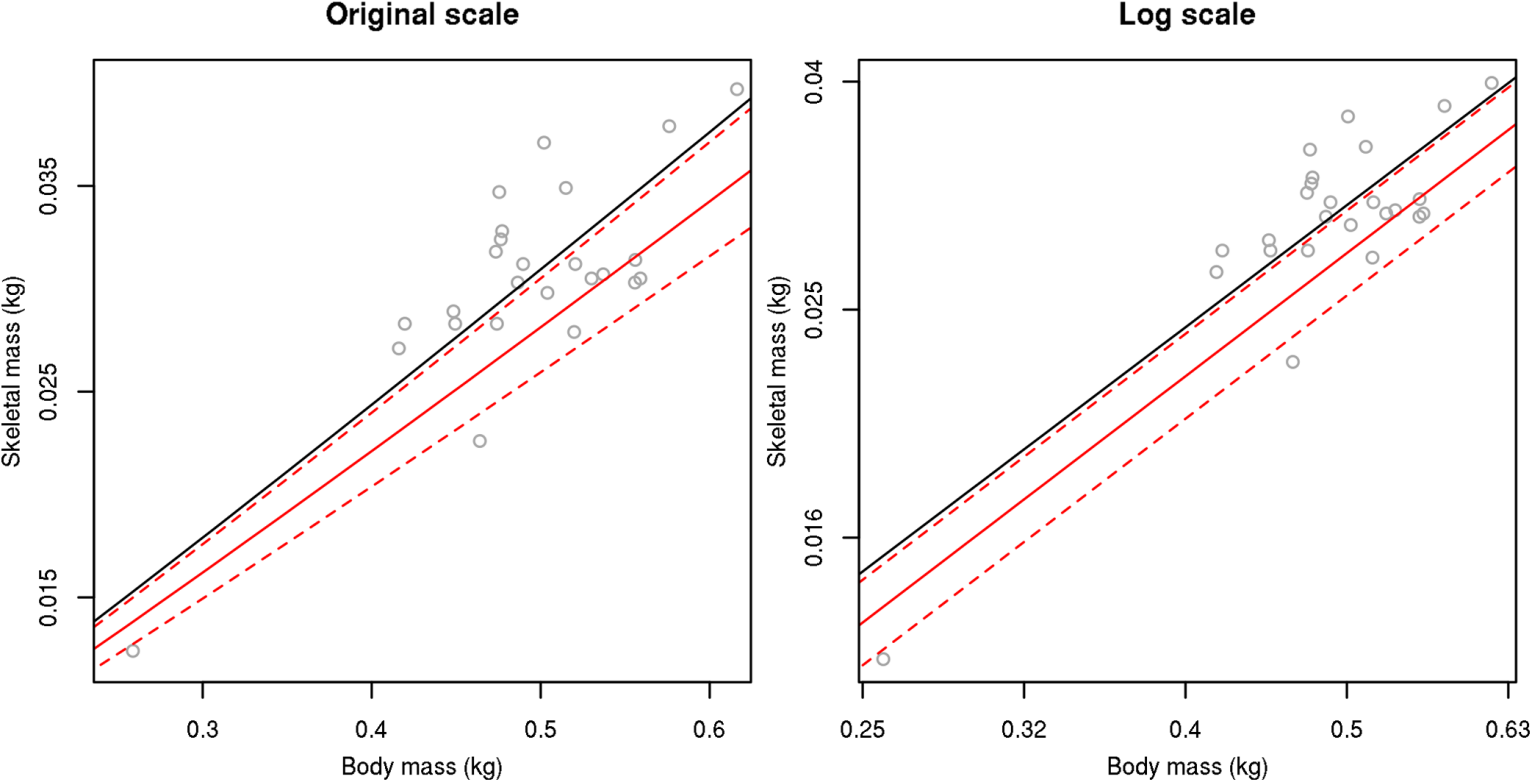
Total body mass and skeletal mass variation in the Rhinoceros Auklet (*Cerorhinca monocerata*) showing within-species variation and deviation from regression slopes. Black line represents the association estimated by Prange et al. [7], red line represents the association estimated here based on the pooled dataset, dashed lines represent associated standard errors.

It is important to further discuss our highly variable new dataset; skeletal mass and TBM are variable within a species. For example, the Rhinoceros Auklet (*Cerorhinca monocerata*) has TBM ranging from 258.7 g to 616.2g, and skeletal masses from 12.4–39.7g (Fig 4). Additionally, the Northern Saw-whet Owl (*Aegolius acadicus*) is even more variable, with ranges from 48–184g TBM and skeletal mass from 3.3–5.7g, with the heaviest individual (TBM = 184g) having a light skeleton (4.4g). These ranges in TBM are primarily due to differences in ages of birds: It can be difficult to tell when found how old the bird is, so some of these may be younger individuals (perhaps non-breeding subadults). Additionally, female TBM will vary as they collect and expend resources during the breeding season, accumulate calcium in bones and mobilise it to create eggshells. Similarly, whole bird TBM is known to fluctuate before and during migration. Looking at *A*. *acadicus* again, in addition to the heaviest animal having a light skeleton, there is a difference in skeletal mass of almost 2g in animals with TBM of 60.4–60.9g (skeletal mass 3.5 and 5.3g, respectively). The skeletal masses of some birds are known to change during moulting [37] and again during breeding season [38].

It is also important to discuss our results in light of possible sexual variation and differences in flight modes. While there was no significant difference between the sexes, there does appear to be a slight difference between males and females which may be caused by two outliers in the male dataset (Fig 3). In the case of males, the two heaviest species in the RBCM dataset (*Phoebastria nigripes*, *Phoebastria_immutabilis* TBM = 6.15 kg and 4.90 kg on average) have much lower SM than predicted by the relationship (SM = 0.2302 kg and 0.1920 kg on average respectively). Only 2 and 1 male specimens were measured for these species respectively, therefore this low skeletal mass with respect to body mass could be sampling error and may originate from the above average body condition of these three specimens measured. It is possible that these two outlier species alter the slope in the case of males. More specimens where sex is identifiable are needed to determine if this slight difference is indeed significant, and focus should be but on larger-bodied species to be able to generalize our results to a wider body size range.

Additionally, data suggest that for active flying birds, there is no significant difference in the relationship between total body mass and skeletal mass across different flight styles. This is not necessarily expected as TBM strongly affects locomotion (e.g. [5,6]) and therefore this relationship may be expected to change between different locomotory strategies. Further studies should look at burst-adapted flyers such as grouse, ptarmigans, and tinamous in order to see if birds that are adapted primarily to life on the ground (but have the ability to fly when pressured) have any differences with respect to skeletal structure and mass. Extending this further could involve looking at ratites and fully terrestrial forms such as kiwis, emus and ostriches, as well as birds that have been modified for life in the water, including penguins and flightless cormorants. Finally, this study was biased towards non-passerine birds, and additional studies should include passerines and other birds known to exhibit intermittent bounding flight (see [25,27]).

Although a clear and strong scaling relationship exists within our data for modern birds (Neornithes), our analyses cannot inform the issue of whether, or not, exponents are reliable for mass estimations of extinct taxa outside of this clade. Although not significantly different in these datasets, phylogenetic control did result in a different scaling exponent than without control for relationships. This dataset does not allow us to test for differences within taxa that are not closely related, for example enantiornithine or hesperornithine birds, non-avian theropod dinosaurs, or pterosaurs. Enantiornithine birds were dominant during the second half of the Mesozoic, living until the end of the Cretaceous, and were mostly fully flying birds. Their forelimb proportions were generally similar to modern neornithines, but their hind-limb proportions were quite different [39]. In addition to these differences, they also shared a distinct shoulder morphology, as well as a higher degree of pneumaticity and extensive appendicular air sacs not seen in modern birds [40–42], suggesting that a direct use of this relationship would not be reliable. Hesperornithines, on the other hand, were diving birds. They differ from most modern birds by having exceptionally large feet, numerous teeth, vestigial wings, and little to no pneumaticity [43]. The morphological dissimilarities between them and modern neornithines also suggests this relationship may not be ideal, which may explain why mass estimates using different methods relying on modern bird studies result in different masses [43].

Moving further into non-avian dinosaurs makes this relationship even less reliable, and other mass estimation methods such as allometric relationships (e.g. [1,44]) and volumetric methods (e.g. [10,45]) are probably better used in this case.

However, this relationship has been applied to mass estimates for pterosaurs, a group outside the avian-dinosaurian line. With knowledge of dry skeletal mass alone, Witton [13] argued that it is possible to extrapolate total body mass of pterosaurs using the same allometric relationship found in Prange et al. [7], as the relationship appeared to be universal. This relationship was applied to pterosaurs as it avoids the difficulties in estimating the volume and density of soft tissue and the extent of air spaces when calculating total body mass [13]. The exact phylogenetic position of pterosaurs is heavily debated (e.g. [46–48]), but they are most often placed as the sister-group to dinosaurs, with Dinosauromorpha and Pterosauria making up the Ornithodira (e.g. [47,49]. This places them quite far away from neornithines in a phylogenetic sense. This is supported by the fact that other animals such as snakes [50] and lizards [51] appear not to follow the originally Prange et al. [7] relationships, and actually differ substantially.

Martin and Palmer [52] found that the bones of pterosaurs were significantly heavier than those predicted by Witton [13], suggesting that this method may not be entirely accurate for pterosaurs. Pterosaurs are significantly morphologically different than birds, having a single extended digit forming the main wing spar, a flexible membrane forming the wing surface, a filamentous integumentary covering of pycnofibres rather than feathers, and many more [53]. Furthermore, it appears that pterosaurs (at least the larger species) were even more highly pneumatized than birds [54], making them substantially different. These morphological differences combined with the results here indicate that phylogeny plays an important role in this relationship and suggests that this relationship should not be used to determine pterosaur body mass, and alternative methods should be sought.

In conclusion, the previously reported relationship between TBM and skeletal mass in extant neornithine birds is confirmed after more than doubling the sample size, although phylogeny does effect the result, suggesting that this relationship may not be accurate in estimating TBM in extinct non-neornithine clades such as non-avian theropod dinosaurs and pterosaurs. Alternatively, variation cannot be explained by factors including sex, ontogenetic stage, or flight mode (excluding burst-adapted flyers). We encourage others to use this large dataset for additional studies and recommend further analyses of these data.

## Supporting Information

S1 Fig Total body mass and skeletal mass association in two different ontogenetic stages of birds. Red represents hatchling year (HY), blue represents above hatchling year (AHY), black line represents the relationship determined here for the entire data set, coloured solid lines represent the relationship for each ontogenetic stage, while dashed lines represent standard errors.
(TIFF)

S2 Fig Total body mass and skeletal mass association in birds of three different flight modes. Blue = soaring, red = flap-gliding, green = continuous flapping. Solid lines represent the association for each flight mode, dashed lines represent standard errors.
(TIFF)

S1 Table Details of RBCM mass specimens. Species names, common names, specimen numbers, sex (M = male, F = female, ? = unkown), age (HY = hatchling year, AHY = above hatchling year), flight mode and source, total and skeletal masses of specimens used from the Royal British Columbia Museum (RBCM) dataset.
(XLSX)

S2 Table Pooled dataset from RBCM and Prange et al. [7]. (XLSX)

S3 Table Whole RBCM dataset. Dataset includes masses and measurements for individual bones/regions where available, not used in this study.
(XLS)
