# Supplementary figures and images for: Exploring the Relationship between Skeletal Mass and Total Body Mass in Birds
Source: PLoS One. 2015 Oct 28;10(10):e0141794. doi: 10.1371/journal.pone.0141794 (PMC4625084; doi:10.1371/journal.pone.0141794)

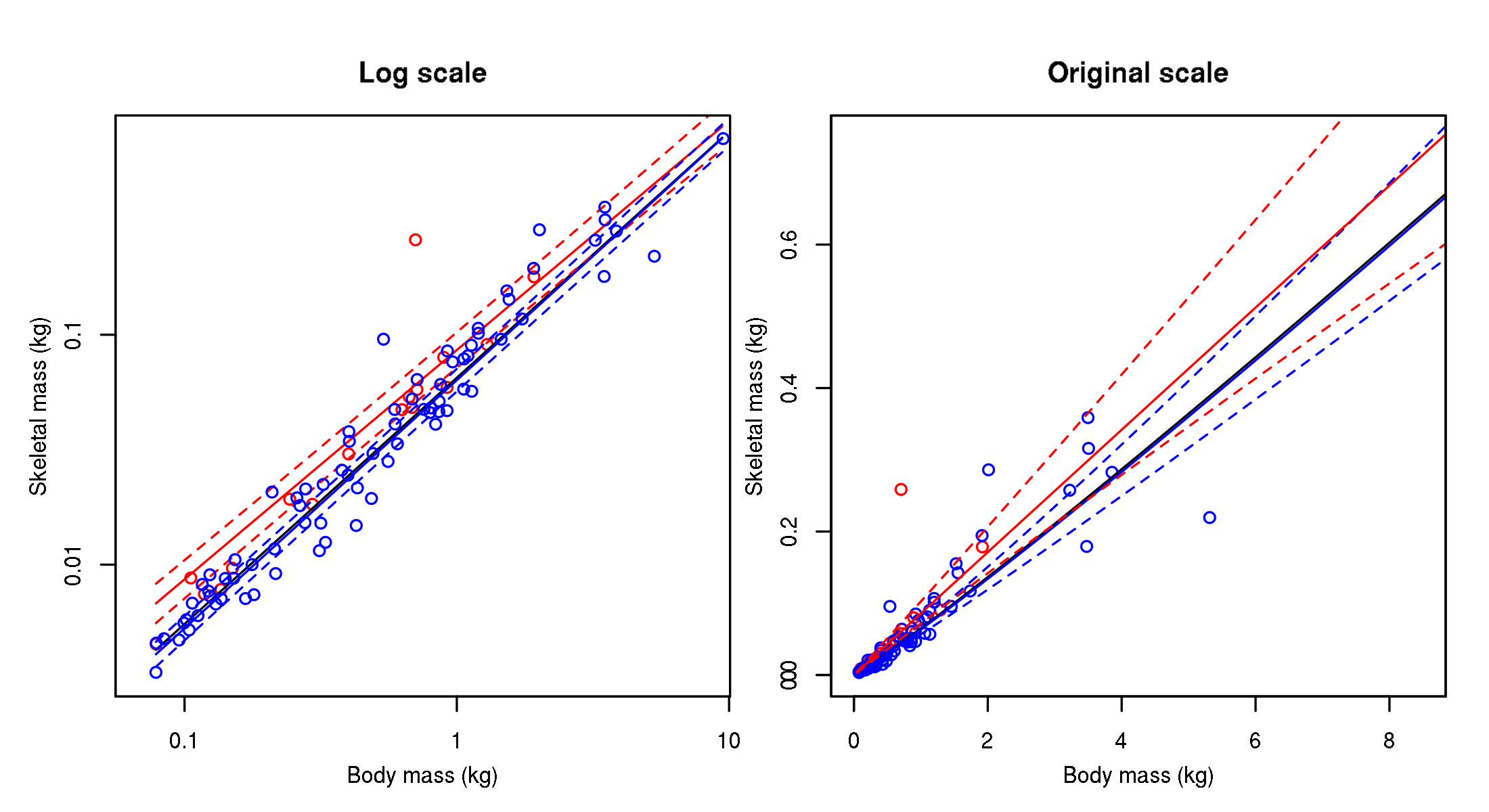

Supplement: S1 Fig — Red represents hatchling year (HY), blue represents above hatchling year (AHY), black line represents the relationship determined here for the entire data set, coloured solid lines represent the relationship for each ontogenetic stage, while dashed lines represent standard errors. (TIFF) [file pone.0141794.s001.tiff]

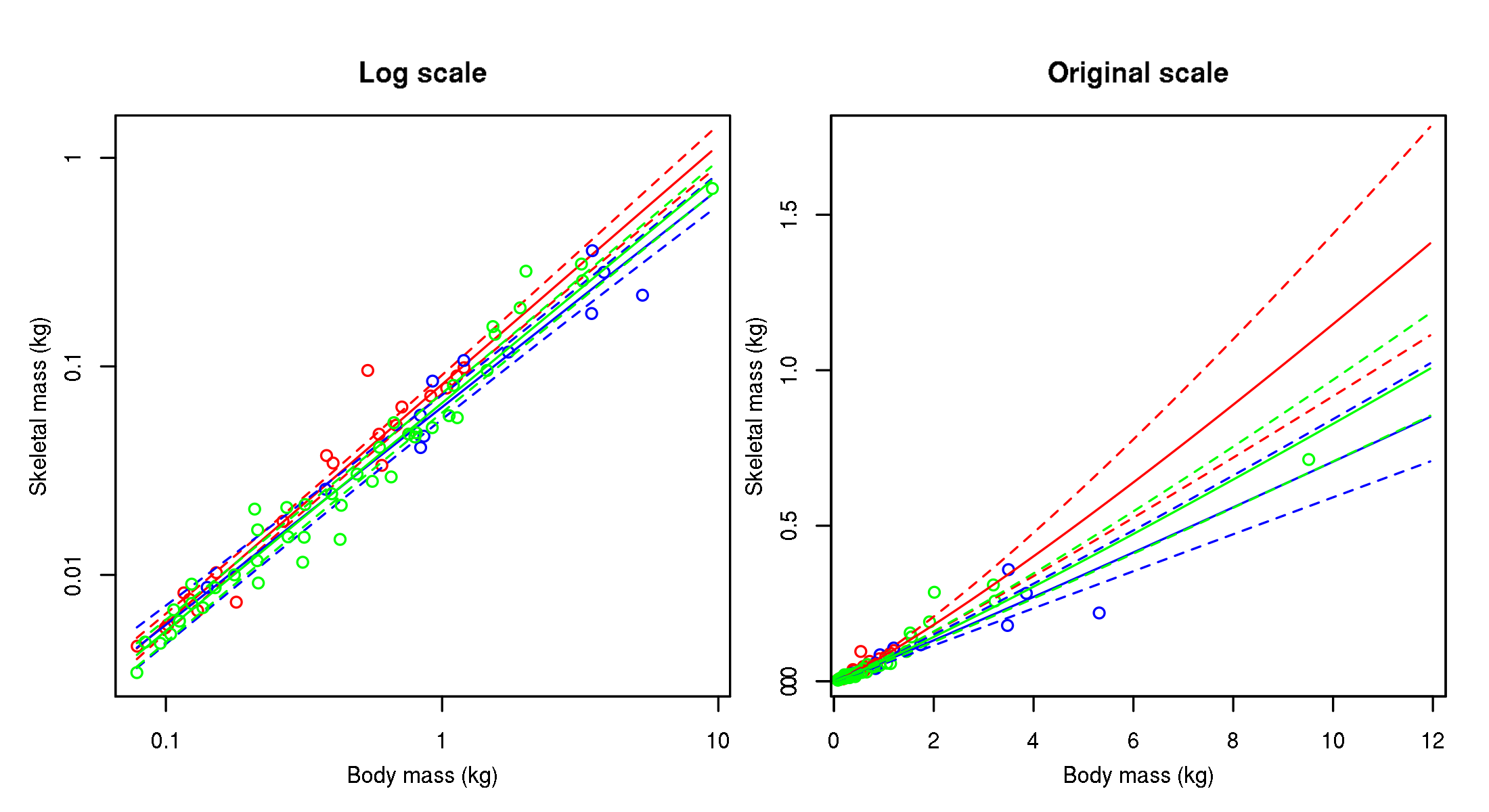

Supplement: S2 Fig — Blue = soaring, red = flap-gliding, green = continuous flapping. Solid lines represent the association for each flight mode, dashed lines represent standard errors. (TIFF) [file pone.0141794.s002.tiff]
